# Restless legs syndrome: literature review

**DOI:** 10.1590/S1516-31802010000300008

**Published:** 2010-05-06

**Authors:** Emmanouil Symvoulakis, Dimitrios Anyfantakis, Christos Lionis

**Affiliations:** I MD, PhD. Scientific collaborator at the Social and Family Medicine Clinic, Department of Social Medicine, School of Medicine, University of Crete, Heraklion, Crete, Greece.; II MD. Doctoral student at the Social and Family Medicine Clinic, Department of Social Medicine, School of Medicine, University of Crete, Heraklion, Crete, Greece.; III MD, PhD. Head of the Social and Family Medicine Clinic, Department of Social Medicine, School of Medicine, University of Crete, Heraklion, Crete, Greece.

**Keywords:** Restless legs syndrome, Primary health care, Classification, Epidemiology, Diagnosis, Síndrome de las piernas inquietas, Atención primaria de salud, Clasificación, Epidemiología, Diagnóstico

## Abstract

Restless legs syndrome is a distressing condition, with negative effects on sleep and daytime activities that affect personal, family and occupational life. The overall impact of restless legs syndrome on quality of life is comparable to that of chronic and frustrating conditions such as depression and diabetes. Misdiagnosis and inappropriate treatment may increase patients’ suffering in terms of uncertainty, overuse or misuse of care services and lack of trust. Presenting a synthesis of the main topics in the literature on restless legs syndrome facilitates for a better understanding and its management in primary care settings.

## RESUMEN

El síndrome de las piernas inquietas representa una condición dolorosa, con efectos negativos en las actividades del sueño y del día, influyendo la vida personal, familiar y profesional. El impacto global del síndrome de las piernas inquietas en la calidad de vida es comparable al de condiciones crónicas y frustrantes como la depresión y la diabetes. Un diagnóstico equivocado y un tratamiento poco adecuado pueden aumentar el sufrimiento del paciente en lo que se refiere a la incertidumbre, la utilización excesiva o no adecuada de los servicios de cuidado y la falta de confianza. El presentación de una síntesis de los tópicos principales de la bibliografía sobre este síndrome facilita su mejor entendimiento y tratamiento en el marco del cuidado médico primario.

## INTRODUCTION

Restless legs syndrome (RLS) is a neurological movement disorder, first described by Sir Thomas Willis in the 17^th^ century.^1^ In the 1940s, Ekbom recognized that this condition leads to significant impact on sleep quality and daytime functional status.^2^ General practitioners (GPs) may face patients who experience severe RLS symptoms more often than other physicians,^3^ since the considerable discomfort caused by their complaints may trigger the use of primary care services. For instance, patients can experience a distressing urge to move the lower limbs. This urge is frequently accompanied by an unpleasant sensation felt deep inside the affected limb which can be described as sensory disorders of "creeping", "crawling", "burning", "pulling" or "soda bubbling in the veins".^4^ The symptoms increase or appear during rest periods, are relieved by movement and mainly occur during periods of inactivity, mostly at night.^4^ For some patients with RLS, the symptoms are mild and cause little inconvenience. However, in others, RLS is a leading cause of sleep disturbances and such individuals’ quality of life can be seriously affected.^5^ RLS can be classified as a primary disease or can arise as a condition secondary to iron deficiency,^6^ pregnancy,^7^ renal failure^8^ or polyneuropathy.^9^

Although the symptoms that are described occur frequently, and patients live their lives experiencing functional impairment and distress, RLS is still underdiagnosed.^3^ Thus, it was of interest to develop a paper that aimed to discuss the need to increase GPs’ clinical familiarity with RLS and enhance their capacities in terms of a more "joined-up" approach to RLS. This could facilitate "powerful" research initiatives within primary care.

## LITERATURE SEARCH

We conducted a literature search in the PubMed database, with limits between 1986 and August 2007, using the MeSH (Medical Subject Headings) term "Restless legs syndrome". The search was limited to studies published in the English language. The search queries used included clinical trials, meta-analyses, practice guidelines, randomized controlled trials, editorials, case reports and reviews. The initial search resulted in 564 articles (Table 1). Papers providing information relevant to primary care use and offering evidence on routine practice issues relating to the disorder were included. Finally, the thematic content and reporting details of the papers included were scrutinized to enable a better approach towards the main topics relating to RLS, such as epidemiology, clinical presentation, diagnosis, management and patients’ quality of life. Historical and additional citations were identified from the reference lists of the publications included and were added to increase the completeness of the information discussed.

**Table 1.** Database search results

Database	Search strategy	Results
PubMed	Restless legs syndrome [MeSH]	225 review articles
		59 randomized controlled trials
		70 clinical trials
		36 editorials
		171 case reports
		3 practice guidelines

MeSH = Medical Subjects Headings.

## CLASSIFICATION

Primary RLS is the most common type of RLS, and affects patients without any other underlying disorder that can explain the reported symptoms. Approximately six out of every ten of these patients have a family history of RLS, which suggests genetic predisposition.^10^ In these patients, the inheritance pattern suggests that RLS is transmitted autosomally.^10^ Once it starts, it usually becomes a lifelong condition with variable clinical evolution.^4,11^ People who experience RLS symptoms at an early age are more likely to have a family history of the disorder than are others who are affected at an older age.^12^

The secondary form refers to RLS that is derived from some medical conditions such as pregnancy, end-stage renal failure, iron deficiency and polyneuropathies. RLS may appear during pregnancy or can be aggravated by this condition. In a study on 500 pregnant women, 19% presented this condition.^7^ This is usually a benign form of RLS, with the highest level of severity in the third trimester and a tendency to disappear after delivery.^13^ Inadequate iron stores can also be associated with RLS. Studies have shown that serum ferritin levels below 50 mcg/l may be related to greater intensity of RLS symptoms. After iron replacement therapy, there is an improvement in the symptoms.^6,14^ Moreover, patients with end-stage renal disease are often affected.^8,15,16^ The reported prevalence of RLS ranges from 20 to 57% in this group of patients. In some cases, RLS can become remittent after renal transplantation.^17^ Polyneuropathies are also associated with RLS. Gemignani et al. found that approximately three out of every ten patients in their cohort with polyneuropathy suffered from RLS.^9^ Small fiber sensory neuropathy was the most frequent occurrence among their RLS patients.^9^

Published data show that some medications can worsen or trigger RLS, such as tricyclic antidepressants,^18^ selective serotonin reuptake inhibitors (SSRIs),^19^ lithium,^20^ dopamine antagonists^21^ and caffeine.^22^

Finally, in the case of diabetes mellitus and Parkinson disease, there is controversy in the literature caused by the lack of evidence supporting any strong connection between these conditions and RLS.^23^

## THE BURDEN OF THE PROBLEM

Towards better recognition of RLS, the International Restless Legs Syndrome Study Group established standard diagnostic criteria in 1995,^24^ which were updated in 2003.^4^ Based on these diagnostic guidelines, recent studies estimated that RLS is a common disorder affecting about 5-10% of the general population in Europe and the United States.^25,26^ A lower prevalence occurs among populations of Asian origin.^27^

A survey conducted in France reported a prevalence rate of 8.5% in the general population.^28^ Furthermore, RLS was diagnosed at a frequency of 7.2% in the REST study involving 15,391 adults in the United States and Europe.^29^ Approximately 3% of the patients reported symptoms at least twice weekly, experiencing moderate to severe distress. It was found that the symptoms in this group of patients had a negative impact on their quality of life, comparable with other serious nosological entities such as type 2 diabetes mellitus and depression. It is remarkable that although many sufferers in this subgroup discussed their symptoms with a primary care physician, RLS was significantly underestimated and misdiagnosed.^29^ GPs were not aware of the presence of this common disorder and the symptoms were wrongly attributed to other more frequent medical conditions like back pain, arthritis, varicose veins, depression and anxiety.^29^

The REST study, conducted among 23,000 people in primary care settings, highlighted the need for better understanding of the condition by GPs, taking into account the high prevalence of the disorder, the significant functional impact and the low rates of diagnosis in primary care settings.^30^ The prevalence among adults whose symptoms were severe enough to report them to their primary care physician was estimated to be over 3%.^30^ Most of them were experiencing both sensory and sleep-related symptoms.^30^ It is noteworthy that approximately nine out of every ten RLS sufferers reported at least one sleep-related symptom.^30^ Almost seven out of ten of these patients reported taking over 30 minutes to get to sleep, and approximately six out of ten woke three or more times per night.^30^

The evidence suggests that RLS has not been managed adequately, even in the rare cases when it was correctly recognized.^30^ GPs’ doubts are reflected in the wide range of diagnoses put forward.^30^ Patients continue to suffer and are inappropriately referred to other specialists for further investigation or treated with medication not suited for RLS.^30^ These data partly reflect the current lack of good medical practice guidelines relating to this disorder. Supporting this idea, a study on United Kingdom primary care clinics covering the period 1994-1998 reported very low prevalence (0.25%) and RLS incidence of 41 per 100,000 person-years.^31^ This finding could be indicative of the lack of awareness of RLS in primary care settings, thereby leading to poor recognition.^31^

## CLINICAL PRESENTATION AND DIAGNOSIS WITHIN PRIMARY CARE

Patients usually consult their physician after noticing several symptoms, such as insomnia, fatigue, distress or pain in the limbs or in other parts of the body.^32^ They may also report depression or anxiety.^32^ Diagnosing RLS is straightforward and can be done in primary care settings by carefully evaluating the clinical symptoms,^33^ and asking assertive questions based on the diagnostic criteria, as updated in 2003.^4^ These diagnostic criteria involve four basic diagnostic features, three supportive features and three associated clinical characteristics (Chart 1).^4^ Supportive and associated clinical features are not necessary to confirm diagnoses. They commonly coexist and may support the diagnosis, especially in ambiguous cases.^4^

**Chart 1.** Diagnostic criteria and supportive and associated features of restless legs syndrome (RLS) based on the report from the restless legs syndrome diagnosis and epidemiology workshop at the National Institutes of Health^4^

**Essential RLS diagnostic criteria** An urge to move the legs, usually accompanied or caused by uncomfortable and unpleasant sensations in the legs.* The urge to move or unpleasant sensations begin or worsen during periods of rest or inactivity such as lying down or sitting. The urge to move or unpleasant sensations are partially or totally relieved by movement (walking or stretching), at least as long as the activity continues. The urge to move or unpleasant sensations are worse in the evening or at night than during the day, or only occur in the evening or night.†
**Supportive RLS features include:** RLS family history. The prevalence of RLS among first-degree relatives is three to five times higher than among those without RLS. Positive response to dopaminergic treatment. Periodic limp movements (during sleep or wakefulness).
**Associated RLS features include:** Natural clinical course. The clinical course of RLS varies considerably. When RLS symptoms start at an age of less than 50 years, the onset is often more insidious. When the onset is at an age of over 50 years, RLS symptoms often occur with greater intensity. Sleep disturbance. Disturbed sleep is a common major morbidity due to RLS and it is often the reason that leads patients to seek medical help. Medical evaluation/physical examination. Medical information is useful in order to determine whether comorbidities or secondary causes of RLS might be present. Patient evaluations regarding iron status and conditions such as peripheral neuropathy and radiculopathy are important, since additional treatment approaches may be required.

* Sometimes the urge to move is present without the uncomfortable sensations and sometimes the arms or other body parts are involved, in addition to the legs.

† When symptoms are very severe, the worsening at night may not be noticeable but needs to have been present previously.

Since diagnosing RLS is relatively straightforward in primary care settings, meticulous evaluation of patients’ symptoms is imperative.^34^ For instance, the DESYR study on primary care reported that spontaneous complaints of sleep or leg symptoms are frequently associated with the diagnosis of RLS.^35^ Despite the significant progress achieved regarding RLS over the past 20 years, current evidence suggests that this very common neurological movement disorder is often overlooked by GPs. Taking into account the possible "clinical uncertainties" relating to RLS diagnosis, we have compiled some key points for increasing diagnostic surveillance in primary care settings, which we present in Chart 2.^4,6-9,30,33,36,37^

**Chart 2.** Key points for facilitating restless legs syndrome (RLS) diagnosis in primary care^4,6-9,30,33,36,37^

Improve general practitioners’ (GPs’) clinical familiarity with RLS Increase GPs’ awareness of diagnostic "red flags" for RLS, such as complaints of augmented sleep latency, frequent waking during the night and paresthesia or dysesthesia of the legs. Rule out a diagnosis of RLS in patients with disorders that trigger the syndrome, such as pregnancy, end-stage renal failure, iron deficiency or polyneuropathy. Distinguish conditions such as depression and anxiety which may disguise RLS symptoms, and vice versa. Recognize the terms that patients use to describe their RLS symptoms. Comprehensive interaction between physician and patient is required.

## TREATMENT

Patients with RLS are divided into three groups: those with intermittent symptoms; those with daily symptoms; and those whose symptoms are refractory to standard treatments.^38^ Non-pharmacological approaches may be sufficient and may be successful for some patients with mild and rare symptoms. Nevertheless, there is a lack of evidence regarding their feasibility and effectiveness within RLS therapeutic management.

### Non-pharmacological approach

One important goal of non-pharmacologic interventions is to improve sleep hygiene, which includes activities that can achieve better sleep habits. Patients must be encouraged to structure a routine sleep schedule, with regular times for going to sleep and waking up.^38^ Disturbing activities such as excessive exercise during the hours immediately preceding bedtime must be avoided.^39^ Sleeping environments must be kept quiet and comfortable.

Lifestyle modifications are also important. Caffeine and nicotine intake can worsen RLS, and so they should be avoided.^38^ Alcohol often exacerbates RLS symptoms.^38^ Medications such as antidepressants (SSRIs or tricyclics), antihistamines, dopamine-blocking agents (neuroleptics or metoclopramide) may cause deterioration of RLS-related symptoms.^38^

Individuals incorporating a moderate level of exercise into their daily routine could experience benefit.^40^ For patients whose symptoms manifest during times of rest, physicians may recommend activities such as involvement in video games, needlework or painting. Discussion of the symptoms may reduce them.^38^ In general, patients with RLS are well advised to follow a healthy lifestyle with a balanced diet and adequate physical activity.^39^

### Pharmacological treatment

Non-pharmacological strategies may not be effective for patients with moderate to severe symptoms. These patients will require some medication to make their symptoms tolerable.

Intermittent symptoms can be managed using medication that can be taken only when symptoms recur, rather than systematically.^38^ The recommended medications include carbidopa/levodopa, (25/100 mg) taken before bedtime; low potency opioids or opioid receptor agonists [codeine (30-60 mg), propoxyphene hydrochloride (65-130 mg) or tramadol (50-100 mg)]; or benzodiazepines [triazolam (0.125-0.5 mg)].^38,41^ Levodopa only has limited use, because of complications such as augmentation and rebound.^38^ Augmentation is defined as the worsening of RLS symptoms, including earlier onset and higher intensity.^42^ Patients need to be warned about this phenomenon and, if it occurs, the drug must be discontinued and replaced by another agent.^38^ Dopamine agonists have a longer onset of action, and are less useful if taken after symptoms develop.^38^

Daily symptoms may require patients to take medications on a daily basis.^38^ The first-line treatment for daily RLS symptoms is dopamine agonists.^43,44^ Non-ergot dopamine agonists [pramipexole (0.125-2 mg day) or ropinirole (0.125-4 mg/day)] are preferred because of their favorable adverse effect profile.^38^ Although augmentation is less common with these drugs, it may occur in long-term treatment with pramipexole.^45^ The alternatives are anticonvulsants or low potency opioids.^38^ Even though reporting that augmentation syndrome was unlikely to occur during short-term follow-up, Conti et al. suggested that physicians should be aware of this eventuality over long-term periods.^46^

Patients with refractory RLS symptoms may require a change of medication.^38^ This may mean the use of a different dopamine agonist, an opioid or an anticonvulsant.^38^ Addition of a second medication such as benzodiazepine, gabapentin or opioid, with reduction of the initial agonist dose, may be helpful.^39^ In the most severe cases, strong opioids such as methadone (5-40 mg/day) have proved useful.^47^

## CONCLUSIONS

It is known that many physicians ignore or wrongly treat RLS.^48^ Helpless patients, attempting to seek relief from their distressing status, consult secondary care specialists like neurologists, psychiatrists, rheumatologists or vascular specialists.^30^ In addition, if and when GPs are "neurophobic", they frequently encourage patients to use or overuse healthcare services. Sometimes, diagnosis and referral pathways are complex issues, and perhaps this is beyond the aim of this paper.^49^ However, in some cases of suspicion of another sleep disorder^50^ or in severe RLS forms refractory to medication, the interface between primary and secondary care might be important. A multidisciplinary approach in which GPs, neurologists, psychiatrists, sleep specialists, and psychologists engage in interactive communication may lead to improved outcomes, especially for patients who experience augmentation or rebound.

Consistent information based on methodologically sound research efforts, critical reading and dissemination of the results needs to be established. This will lead GPs to improve their clinical performance, regarding assessment of which RLS patients are the best candidates for primary care management and when RLS referrals are really "medically explained events". This is more challenging than ever, especially in countries such as Greece, where primary care is still facing intrinsic controversies and extrinsic barriers.

## References

[1] Willis T (1685). The London practice of physick.

[2] Ekbom KA (1945). Restless legs. Acta Med Scand Suppl.

[3] Nichols DA, Allen RP, Grauke JH (2003). Restless legs syndrome symptoms in primary care: a prevalence study. Arch Intern. Med.

[4] Allen RP, Picchietti D, Hening WA (2003). Restless legs syndrome: diagnostic criteria, special considerations, and epidemiology.

[5] Abetz L, Allen R, Follet A (2004). Evaluating the quality of life of patients with restless legs syndrome. Clin Ther.

[6] Sun ER, Chen CA, Ho G, Earley CJ, Allen RP (1998). Iron and the restless legs syndrome. Sleep.

[7] Goodman JD, Brodie C, Ayida GA (1988). Restless leg syndrome in pregnancy. BMJ.

[8] Winkelman JW, Chertow GM, Lazarus JM (1996). Restless legs syndrome in end-stage renal disease. Am J Kidney Dis.

[9] Gemignani F, Brindani F, Negrotti A (2006). Restless legs syndrome and polyneuropathy. Mov Disord.

[10] Montplaisir J, Boucher S, Poirier G (1997). Clinical, polysomnographic, and genetic characteristics of restless legs syndrome: a study of 133 patients diagnosed with new standard criteria. Mov Disord.

[11] Rodrigues RN, Rodrigues AA, Faber J, Corso JT, Peixoto TF (2009). Evolution of non-treated restless legs syndrome. Arq Neuropsiquiatr.

[12] Winkelmann J, Wetter TC, Collado-Seidel V (2000). Clinical characteristics and frequency of the hereditary restless legs syndrome in a population of 300 patients. Sleep.

[13] Manconi M, Govoni V, De Vito A (2004). Restless legs syndrome in pregnancy. Neurology.

[14] Aul EA, Davis BJ, Rodnitzky RL (1998). The importance of formal serum iron studies in the assessment of restless legs syndrome. Neurology.

[15] Gigli GL, Adorati M, Dolso P (2004). Restless legs syndrome in end-stage renal disease. Sleep Med.

[16] Goffredo GS, Gorini CC, Purysko AS, Silva HC, Elias IE (2003). Restless legs syndrome in patients on chronic hemodialysis in a Brazilian city: frequency, biochemical findings and comorbidities. Arq Neuropsiquiatr.

[17] Yasuda T, Nishimura A, Katsuki Y, Tsuji Y (1986). Restless legs syndrome treated successfully by kidney transplantation--a case report. Clin Transpl.

[18] Garvey MJ, Tollefson GD (1987). Occurrence of myoclonus in patients treated with cyclic antidepressants. Arch Gen Psychiatry.

[19] Bakshi R (1996). Fluoxetine and restless legs syndrome. J Neurol Sci.

[20] Terao T, Terao M, Yoshimura R, Abe K (1991). Restless legs syndrome induced by lithium. Biol Psychiatry.

[21] Ward NG (1989). Akathisia associated with droperidol during epidural anesthesia. Anesthesiology.

[22] Lutz EG (1978). Restless legs, anxiety and caffeinism. J Clin Psychiatry.

[23] Garcia-Borreguero D, Egatz R, Winkelmann J, Berger K (2006). Epidemiology of restless legs syndrome: the current status. Sleep Med Rev.

[24] Walters AS (1995). Toward a better definition of the restless legs syndrome. The International Restless Legs Syndrome Study Group. Mov Disord.

[25] Berger K, Luedemann J, Trenkwalder C, John U, Kessler C (2004). Sex and the risk of restless leg syndrome in the general population. Arch Intern Med.

[26] Zucconi M, Ferini-Strambi L (2004). Epidemiology and clinical findings of restless legs syndrome. Sleep Med.

[27] Tan EK, Seah A, See SJ (2001). Restless legs syndrome in an Asian population: A study in Singapore. Mov Disord.

[28] Tison F, Crochard A, Léger D (2005). Epidemiology of restless legs syndrome in French adults: a nationwide survey: the INSTANT Study. Neurology.

[29] Allen RP, Walters AS, Montplaisir J (2005). Restless legs syndrome prevalence and impact. REST general population study. Arch Intern Med.

[30] Hening W, Walters AS, Allen RP (2004). Impact, diagnosis and treatment of restless legs syndrome (RLS) in a primary care population: the REST (RLS epidemiology, symptoms, and treatment) primary care study. Sleep Med.

[31] Van De Vijver DA, Walley T, Petri H (2004). Epidemiology of restless legs syndrome as diagnosed in UK primary care. Sleep Med.

[32] Earley CJ (2003). Clinical practice. Restless legs syndrome. N Engl J Med.

[33] Chaudhuri KR, Forbes A, Grosset D (2004). Diagnosing restless legs syndrome (RLS) in primary care. Curr Med Res Opin.

[34] Wellbery CE (2000). Getting the facts on restless legs. Am Fam Physician.

[35] Crochard A, El Hasnaoui A, Pouchain D (2007). Diagnostic indicators of restless legs syndrome in primary care consultations: the DESYR Study. Mov Disord.

[36] Wittmaack T (1861). Pathologie und therapie der sensibilität-neurosen.

[37] Masuko AH, Carvalho LB, Machado MA (2008). Translation and validation into the Brazilian Portuguese of the restless legs syndrome rating scale of the International Restless Legs Syndrome Study Group. Arq Neuropsiquiatr.

[38] Silber MH, Ehrenberg BL, Allen RP (2004). An algorithm for the management of restless legs syndrome. Mayo Clin Proc.

[39] Hening WA (2007). Current guidelines and standards of practice for restless legs syndrome. Am J Med.

[40] Aukerman MM, Aukerman D, Bayard M (2006). Exercise and restless legs syndrome: a randomized controlled trial. J Am Board Fam Med.

[41] Spolador T, Allis JC, Pondé MP (2006). Treatment of restless legs syndrome. Rev Bras Psiquiatr.

[42] Earley CJ, Allen RP (1996). Pergolide and carbidopa/levodopa treatment of the restless legs syndrome and periodic leg movements in sleep in a consecutive series of patients. Sleep.

[43] Hening WA, Allen RP, Earley CJ, Picchietti DL, Silber MH (2004). Restless Legs Syndrome Task Force of the Standards of Practice Committee of the American Academy of Sleep Medicine. An update on the dopaminergic treatment of restless legs syndrome and periodic limb movement disorder. Sleep.

[44] Trenkwalder C, Garcia-Borreguero D, Montagna P (2004). Ropinirole in the treatment of restless legs syndrome: results from the TREAT RLS 1 study, a 12 week, randomised, placebo controlled study in 10 European countries. J Neurol Neurosurg Psychiatry.

[45] Winkelman JW, Johnston L (2004). Augmentation and tolerance with long-term pramipexole treatment of restless leg syndrome (RLS). Sleep Med.

[46] Conti CF, Oliveira MM, Valbuza JS (2008). Anticonvulsants to treat idiopathic restless legs syndrome: systematic review. Arq Neuropsiquiatr.

[47] Ondo WG (2005). Methadone for refractory restless legs syndrome. Mov Disord.

[48] O’Keeffe ST, Egan D, Myers A, Redmond S (2007). The frequency and impact of restless legs syndrome in primary care. Ir Med J.

[49] Vinker S, Kaiserman I, Karni A (2007). Urgent referrals to a specialist by family physicians--is the "urgency" real: a prospective study. Eur J Gen Pract.

[50] Rodrigues RN, Abreu e Silva Rodrigues AA, Pratesi R (2007). Outcome of sleepiness and fatigue scores in obstructive sleep apnea syndrome patients with and without restless legs syndrome after nasal CPAP. Arq Neuropsiquiatr.

